# Efficacy and Safety of Traditional Chinese Medicine on Nonerosive Reflux Disease: A Meta-Analysis of Randomized Controlled Trials

**DOI:** 10.1155/2018/1505394

**Published:** 2018-05-24

**Authors:** Jiao Xiao, Yunfeng Yang, Yuanrong Zhu, Yan Qin, Yifan Li, Mengjie Fu, Zhengdong Zhai, Lingyun Zhu

**Affiliations:** ^1^Shanghai Municipal Hospital of Traditional Chinese Medicine Affiliated to Shanghai University of Traditional Chinese Medicine, Shanghai 200071, China; ^2^Fenglin Community Health Service Center of Xuhui District, Shanghai 200030, China; ^3^Xujiahui Community Health Service Center of Xuhui District, Shanghai 200030, China; ^4^Xiaoshan Traditional Chinese Medical Hospital, Hangzhou 311201, China; ^5^Yingpu Community Health Service Center of Qingpu District, Shanghai 201799, China

## Abstract

**Objectives:**

Traditional Chinese medicine (TCM) therapy for nonerosive reflux disease (NERD) remains controversial. The aim of this study was to evaluate the efficacy and safety of TCM regimens in NERD treatment.

**Methods:**

Randomized controlled trials (RCTs) of TCM treatment for NERD through September 31, 2017, were systematically identified in PubMed, Wanfang Data, CNKI, VIP, CBM, Ovid, Web of Science, and Cochrane Library databases. Quality assessment was performed by employing the Cochrane Risk of Bias assessment tool.

**Results:**

A total of 725 and 719 patients in 14 RCTs were randomly divided into TCM alone and conventional Western medicine groups, respectively. The clinical total effective rate of the TCM group was markedly higher than that of the single proton pump inhibitors (PPIs) or Prokinetics therapy group (RR = 1.19, 95% CI = 1.07–1.31, and *P* = 0.0008), while it was comparable to that of the combination of PPIs and Prokinetics therapy group (RR = 1.14, 95% CI = 1.00–1.29, and *P* = 0.05). Compared with Western medicine group, the TCM group showed improved symptom relief through a reduced RDQ score (SMD = −0.91; 95% CI = −1.68–−0.15; and *P* = 0.02). Additionally, TCM clearly decreased the recurrence rate (RR = 0.38, 95% CI = 0.28–0.52, and *P* < 0.00001). Adverse events, such as constipation, sickness, fever, abdominal distension, and stomach noise, were slight for both the TCM and Western medicine groups and disappeared after the easement of pharmacological intervention; in particular, TCM possessed fewer side effects.

**Conclusion:**

Compared with PPIs or Prokinetics therapy alone, TCM single therapy can better improve the clinical total effective rate and symptom relief and decrease the recurrence rate and adverse events in the treatment of NERD. Our results suggest that TCM will be a promising alternative therapy for NERD patients in the future.

## 1. Introduction

Nonerosive reflux disease (NERD) has generally been defined as the existence of typical symptoms such as regurgitation, heartburn, or chest pain without upper endoscopy esophageal mucosa injury, which is also known as endoscopic negative reflux disease or symptomatic gastroesophageal reflux disease (GERD) [1]. NERD is the most widespread phenotype of GERD. Epidemiological studies have shown that the morbidity of GERD is close to 20%–40% in Western countries [1] and is 5% to 17% in Asian countries [2], and the incidence of GERD worldwide is increasing. The results of various epidemiological studies have shown that the prevalence of NERD in the GERD population is between 50% and 70% [3].

Proton pump inhibitors (PPIs) are recognized as the first-line drug for NERD. Several clinical trials have confirmed that PPIs are less effective at relieving heartburn symptoms in patients with NERD than in patients with erosive esophagitis (EE), but PPIs are superior to H_2_ receptor antagonists (H_2_RA) and Prokinetic agents in improving symptoms [4]. Furthermore, two-thirds of NERD patients will demonstrate symptomatic relapse from PPIs over time [5], seriously affecting the quality of life of patients.

In recent years, increasing clinical studies have indicated that traditional Chinese medicine (TCM) has more advantages for NERD than PPIs or other Prokinetics [6]. Abundant evidence has demonstrated that Chinese herbal prescriptions or compounds with various types of medicinal ingredients can substantially alleviate symptoms, apparently reduce the incidence rate of adverse events, and lower the recurrence rate [7, 8].

Regrettably, reports of randomized controlled trials (RCTs) on the treatment of NERD with TCM are still in poor quality, dissatisfying the CONSORT and TREND statement [23]. Many RCTs are presented as randomized trials, but they do not utilize specific random methods and are therefore not truly randomized. Hence, we included only RCTs in our meta-analysis that indicated concrete methods. The aim of the study was to systematically review and meta-analyze data from related RCTs reported both at home and abroad to evaluate the efficacy and safety of TCM against NERD, providing a reference for the clinical and rational usage of drugs and individual treatment.

## 2. Materials and Methods

### 2.1. Search Strategy and Study Selection

We searched PubMed, Wanfang Data, China National Knowledge Infrastructure (CNKI), Chinese Science and Technology Periodical Database (VIP), Chinese Biomedical (CBM), Ovid, Web of Science, and Cochrane Library databases through September 31, 2017. The following search terms for the literature search were used: (“non-erosive reflux disease” OR “NERD”) AND (“traditional Chinese medicine” OR “alternative medicine” OR “complementary medicine” OR “Chinese herbal medicine” OR “herb/herbal” OR “decoction/formulation/granule/pill/pulvis/method”) AND (“clinical study” OR “clinical trial” OR “randomized controlled trial” OR “randomized controlled trial”). The search was carried out mainly in Chinese and English. We manually searched reference lists of all review articles, major studies, and abstracts from meetings after the electronic search to identify other studies not found in the electronic search. Two investigators (J. Xiao and Y. Yang) independently searched the eligible literature and extracted data. When a disagreement between two investigators occurred, it was settled by discussion.

The systematic review was conducted on the basis of the Preferred Reporting Items for Systematic Review and Meta-Analyses Statement (PRISMA) [24]. Articles that satisfied the following criteria were included: (1) for participants, NERD patients between 18 and 75 years old that met the guidelines or consensus views for GERD and the number per group had to be no less than 15 cases; (2) for study types, RCTs with a concrete randomized method whether or not they were blinded; (3) for interventions, the TCM group was treated with TCM alone, and the control group was treated with individual PPIs or Prokinetics alone or a combination of PPIs and Prokinetics; (4) for outcomes, the total effective rate was used as the primary outcome by referring to guidelines or consensus views or the evaluation criteria of the Guidelines of Clinical Research of New Drugs of Traditional Chinese Medicine (Table 1); and one or all of the following outcome measurements had to be equipped as the secondary outcome in two groups: Reflux Disease Questionnaire (RDQ) scores after treatment, the relapse rate, and adverse events; (5) the baseline data of the two groups before treatment were not statistically significant; and (6) full texts and data were available. If the sources and treatment protocol of the study population enrolled overlapped by more than 30% in two or more reviews by the same author, we included only the most recent studies or studies with more NERD patients. Studies were excluded if they met the following criteria: (1) studies without specific randomized methods; (2) the experimental group received the combination treatment of TCM and Western medicine or a combination TCM with acupoint injection or acupuncture; (3) studies did not have control groups, or control subjects received TCM treatment including herbal medicine, acupuncture, or acupoint injection therapy; (4) studies reported only laboratory indexes and/or each symptom improvement rate rather than the total effective rate.

### 2.2. Data Extraction

The following contents were individually extracted from each included study by two researchers (J. Xiao and Y. Yang): publication data (first author's last name, year of publication, sex, and age); sample size; treatment protocol (TCM name, Western medicine name, and dose); duration of treatment; main outcomes; and random methods. Disagreements were resolved by discussion or consensus with a third reviewer (L, Zhu). We contacted the corresponding author by telephone, email, or fax to acquire the correct data if a study was incomplete or unsure.

### 2.3. Methodological Quality Assessment

The methodological qualities of the included RCTs were assessed according to Cochrane Collaboration's Tool delineated in Handbook version 5.3.0 [25]. Two authors (Jiao Xiao and Yunfeng Yang) separately assessed quality by evaluating the risk of bias, including random sequence generation (selection bias), allocation concealment (selection bias), blindness of participants, personnel (performance bias), incomplete outcome data (attrition bias), selective reporting (reporting bias), and other biases. The quality of RCTs was classified into low bias risk, high bias risk, and unclear bias risk. In the case of discrepancies, other reviewer authors (Yuanrong Zhu and Lingyun Zhu) acted as arbiters in discussions to resolve these disagreements.

### 2.4. Statistical Methods

Data were handled according to the Cochrane Handbook [26]. TCM and Western medicine therapies were compared in this study. All the outcomes, including the clinical total effective rate, the RDQ score, and the relapse rate and adverse events after treatment, were contrasted between the two groups. We divided patients into improved or unimproved according to the authors' own criteria in each study. Furthermore, we deliberated that the results of patients free of symptoms and patients with improved symptoms were equivalent and that each outcome of interest based on a priori expectation of similar size and orientation of therapeutic effects was combined. Therefore, we classified “being cured, markedly effective, and effective” as a positive result and “no improvement” as a negative result.

The measures of clinical effects were the risk ratios (RRs) and the associated 95% confidence intervals (CIs) for dichotomous data, which of RDQ and symptoms total integral were standardized mean differences (SMDs) and 95% CIs for continuous data. The data were merged according to the Mantel-Haenszel (fixed-effects) model and the DerSimonian and Laird (random-effects) model [27]. We evaluated heterogeneity among studies by visually inspecting forest plots and then formally assessed it by Cochrane's *P* values, *I*^2^ tests, and chi-square tests to perform inferences regarding the null hypothesis of homogeneity (considered significant at *P* < 0.10). A coarse guide to our interpretation of *I*^2^ follows:

- 0% to 40% indicates that heterogeneity may not be important.
- 30% to 60% corresponds to mild heterogeneity.
- 50% to 90% shows substantial heterogeneity.
- 75% to 100% means abundant heterogeneity [25, 28].

If the eligibility of certain studies in the meta-analysis was uncertain due to the lack of information, a sensitivity analysis was implemented by meta-analyzing twice: in the original meta-analysis, all studies were included, while only absolute qualified researches were included in the second meta-analysis. The fixed-effects model was primarily used for our meta-analysis; then the stochastic-effects model was employed in the presence of heterogeneity. The analysis was described when the quantitative data could not be aggregated. All the statistical tests were two tailed, and differences were statistically significant at *P* < 0.05. Review Manager Software Version 5.3 (Cochrane Community, London, United Kingdom, 2014) was employed in our data analysis.

## 3. Results

### 3.1. Study and Patient Characteristics

**Figure 1:**
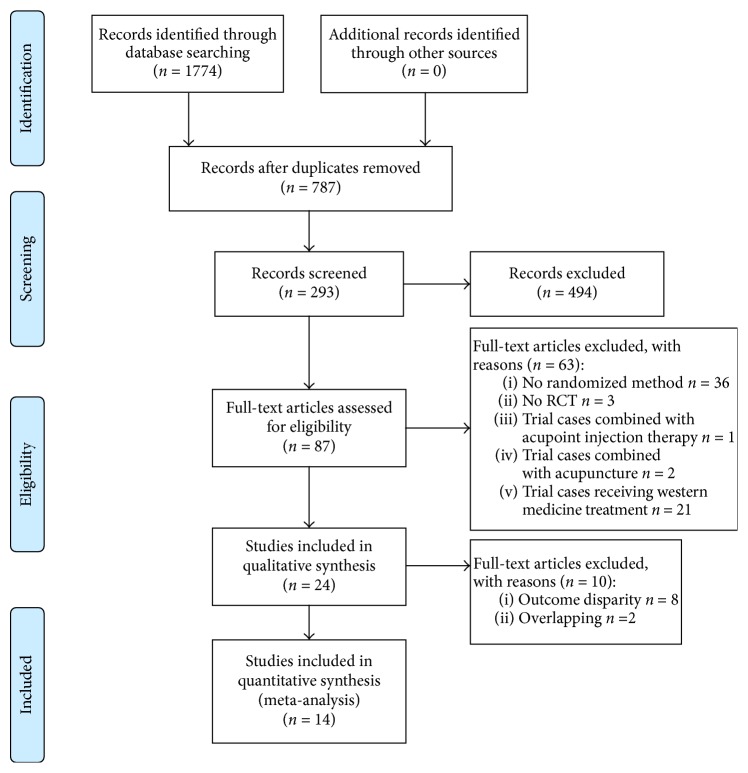
Flow program of study selection.* From*: Moher D, Liberati A, Tetzlaff J, Altman DG, The PRISMA Group (2009). Preferred Reporting Items for Systematic Reviews and Meta-Analyses: The PRISMA Statement. PLoS Med 6(7): e1000097. doi:10.1371/journal.pmed1000097. For more information, visit http://www.prisma-statement.org.

A total of 1774 abstracts were reviewed. Among these reviewed abstracts, 87 articles were retrieved, and 39 were related to the current subject but were ultimately eliminated because they did not include particular random methods or only used simple stochastic method, and 24 were excluded for receiving a combination of TCM and Western medicine or acupuncture therapy in the trial group. Thus, 24 RCTs were relevant to the current theme. However, 8 were excluded because the outcomes were symptom remission rate and TCM syndrome effective rate rather than clinical total effective rate, and 2 overlapped. The specific article selection process is summarized in Figure 1. Finally, 14 RCTs involving 1444 patients with NERD were included on the basis of our inclusion criteria. The baseline characteristics of the included studies are detailed in Table 2.

### 3.2. Methodological Quality Assessment

**Figure 2:**
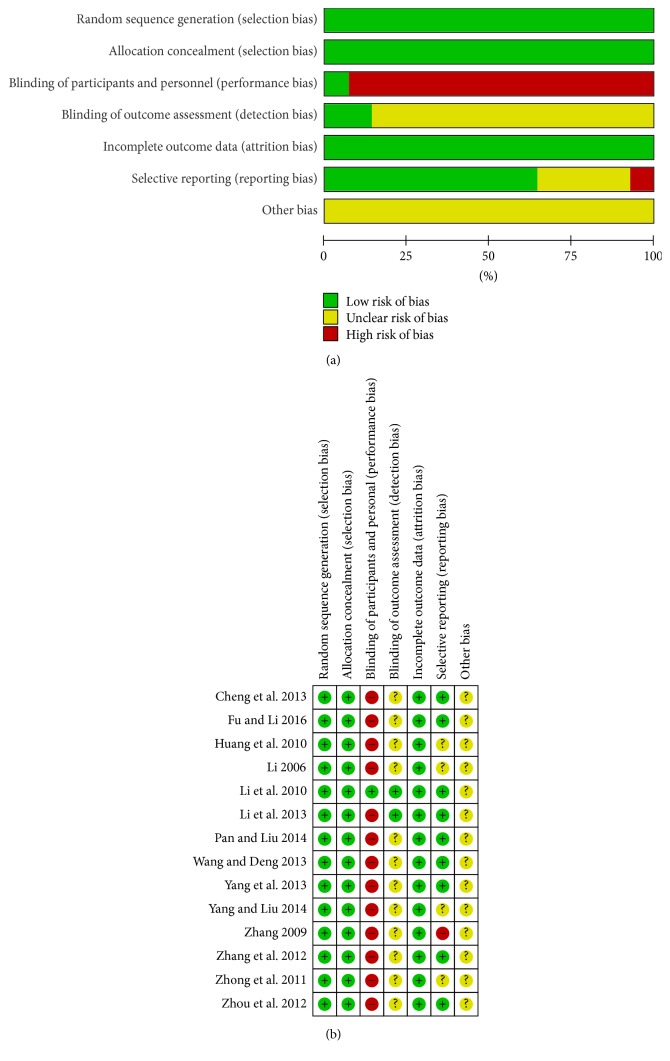
Risk of bias graph (a) and risk of bias summary (b).

The randomized methods were described in detail in all studies [9–22], which were considered as randomized blocks [13], sealed envelopes [12, 14, 20], and random number tables [9, 10, 14–19, 21, 22]. Hence, we considered them low risk in the aspect of selection bias. Almost none of the studies reported blinded methods, which were regarded as high risk in terms of performance bias except for the study reported by Li et al. [13]. Detection bias was low risk in two studies [13, 14], but it was unclear in other studies [9–12, 15–22] in the absence of the blinding of outcomes assessment. In all studies, fewer than 10% of participants dropped out or were lost to follow-up, which was deemed to be low risk in the matter of incomplete outcome data. One study [19] selectively reported partial follow-up cases due to lack of research time, which was considered high risk; four studies [11, 12, 18, 21] were unclear, and other studies [9, 10, 13–17, 20, 22] were low risk. Meanwhile, the risks of these studies were unclear in other biases (Figure 2).

### 3.3. Clinical Total Effective Rates

**Figure 3:**
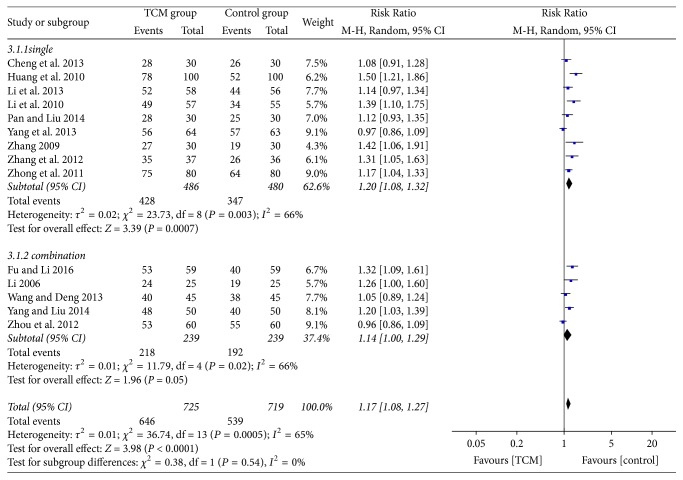
Comparison of a single use of PPIs or Prokinetics (3.1.1) and a combination use of PPIs and Prokinetics (3.1.2) in the total effective rate between two groups.

Seven RCTs [9, 11, 14, 15, 17, 20, 21] reported that the control group was treated with PPIs alone, one was treated RCT [13] with only Prokinetics, and the other five RCTs [10, 12, 16, 18, 22] dealt with PPIs combined with Prokinetics therapy. Therefore, we conducted subgroup analyses for single and combined treatment in the control group. The heterogeneity was substantial when we contrasted the single therapy and combination therapy (*P* = 0.003, *I*^2^ = 66% and *P* = 0.02, *I*^2^ = 66%, resp.). We selected a random-effects model and found that the clinical effects in the TCM group differed significantly from the single PPIs or Prokinetics therapy groups, and no differences existed between the TCM and combination therapy groups (RR = 1.20, 95% CI = 1.08–1.32, and *P* = 0.0007 and RR = 1.14, 95% CI = 1.00–1.29, and *P* = 0.05, resp., Figure 3 (3.1 and 3.2)). Overall, no differences were present by subgroup analysis (*P* = 0.54, *I*^2^ = 0%).

### 3.4. RDQ Scores

**Figure 4:**
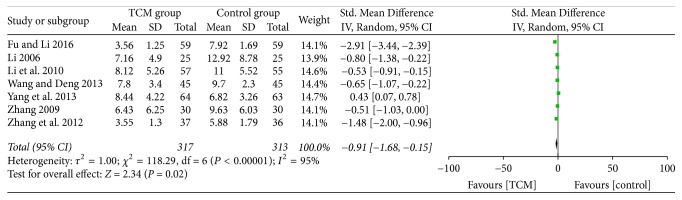
Comparison of the RDQ score after treatment between the TCM and control groups.

We identified five studies [10, 16, 17, 19, 20] that reported RDQ integral modification according to the RDQ scale. We synthesized data of these studies and employed SMDs to eliminate the discrepancy among the studies. Considerate heterogeneity was found (*P* < 0.00001, *I*^2^ = 95%); thus, we used a random-effects model, and the RDQ score in the TCM group was significantly lower than that in the Western medicine group (SMD = −0.91; 95% CI = −1.68–−0.15; and *P* = 0.02, Figure 4).

### 3.5. Recurrence Rates

**Figure 5:**
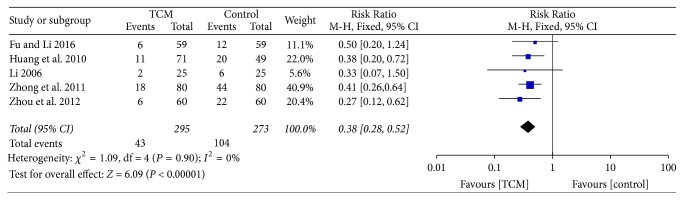
Comparison of the recurrence rate after stopping treatment for more than 3 months between two groups.

Five studies [10–12, 21, 22] reported a relapse rate of more than three months. Our meta-analysis indicated that no heterogeneity (*P* = 0.90, *I*^2^ = 0%) was found, and a striking difference (RR = 0.38, 95% CI = 0.28–0.52, and *P* < 0.00001, Figure 5) was found between the TCM and control group by using a fixed-effects model.

### 3.6. Adverse Events

Eight RCTs reported adverse events in the two groups [10, 11, 13–15, 19, 21, 22]. Three trials did not mention significant adverse reactions (3/8, 37.5%) [11, 14, 19], while five trials described slight discomfort (5/8, 62.5%) [10, 13, 15, 21, 22] that healed themselves after a few days or after adjusting for medication time. In addition, slight discomfort almost occurred in the control group. In two trials [10, 22], two patients showed constipation (2/59, 3.38%) and faint headache (2/59, 3.38%) in the control group. In two trials [10, 15], three participants displayed nausea (3/36, 8.33%). In two trials [15, 21], eight patients exhibited headache (8/56, 14.17%). In one trial [21], two patients manifested dizziness (2/80, 2.5%) and weakness (2/80, 2.5%). In one trial [10], one patient in the TCM group experienced sickness (1/59, 1.69%) and was relieved after adjusting the medication time. In another trial [13], one patient in the TCM group had a cold (1/57, 1.75%) that healed after six days. According to other studies, adverse events of PPIs were reported in 13% of the population, typically in the form of headaches, dizziness, nausea, abdominal pain, diarrhoea, dyspepsia, and flatulence [29]. Relatively speaking, TCM was safer than Western medicine.

### 3.7. Publication Bias

**Figure 6:**
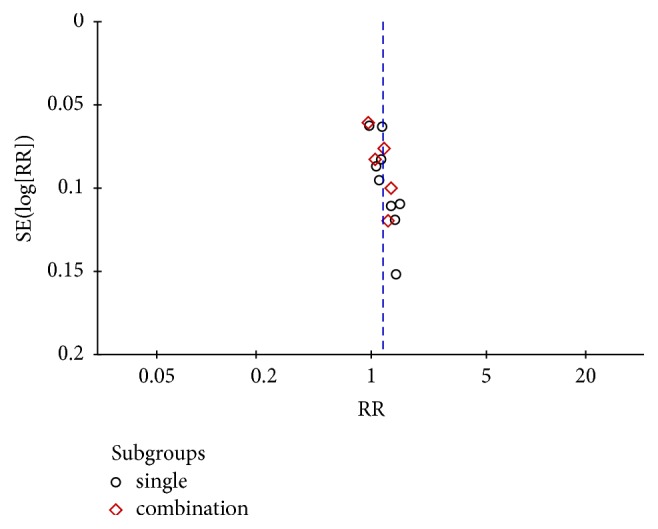
Funnel plot of the comparison of TCM versus Western medicine for the outcome of the total effectiveness rate.

To detect possible publication bias, we analyzed the funnel plot of the 14 trials that compared TCM with Western medicine in terms of the total effective rates. When the total effective rates were pooled, a random-effects model was used.  Figure 6 shows an asymmetrical but centralized funnel plot that indicates publication bias in the 14 selected articles.

## 4. Discussion

In recent years, the incidence rate of NERD has increased, seriously affecting patients' quality of life. The efficacy of PPIs is unsatisfactory for patients with NERD, as they result in a high recurrence rate and side effects and require long-term medication due to the acidic and nonacidic reflux that accompany NERD. Additionally, NERD is often accompanied by anxiety and depression, which immensely affect the efficacy of PPIs. As reported in a meta-analysis, the overall rate of symptomatic relief of PPIs against NERD was 51.4% and the recurrence rate was 51.3% in patients with NERD [30]. A systematic review proposed that on-demand therapy with PPIs is effective at improving symptoms in NERD patients following long-term administration [31]. Moreover, the long-term use of PPIs can lead to many side effects such as bone fractures, community-acquired pneumonia, acute and chronic renal disease, and* Clostridium difficile* intestinal infection [1]. Prokinetics are agents that can promote gastric emptying, enhance esophageal peristalsis and augment lower esophageal sphincter pressure (LESP). However, a meta-analysis indicated that, compared with a single use of PPIs, PPIs combined with Prokinetics therapy had no significant effect on symptoms or endoscopic response of GERD and had increased side effects; however, it may partially improve patients' quality of life [32]. Prokinetics often act as adjuvants in PPI treatment, helping to improve the curative effect and patients' quality of life. Therefore, to improve the clinical effect of patients with NERD, it is urgent to seek a safe and curative alternative therapy.

Although GERD and NERD have not been found in the ancient Chinese literature, the typical symptoms of GERD, “heartburn” and “regurgitation”, are widely documented in the ancient Chinese medical literature. For example, The Yellow Emperor's Inner Classic (Huáng Dì Nèi Jīng), a most classical Chinese medicine theory monograph during the Warring States Period (457–221 BC), first recorded the term “regurgitation”. With the long-term clinical experience of ancient TCM physicians, they found that the aetiological agent of regurgitation is emotional disturbance, and the pathogenesis is closely associated with disharmony between the liver and stomach (zang-fu organs in TCM) [33]. Several studies have reported that the disharmony between the liver and stomach syndrome is the most common syndrome observed in patients with GERD and is accompanied by the highest incidence of psychological problems [34–37]. Moreover, patients who possess the syndrome exhibit a lower quality of life [38]. In a clinical trial, Shugan Hewei Decoction reduced the self-rating anxiety scale scores and self-rating depression scale scores after treatment compared with omeprazole. At the same time, animal experiments found that Shugan Hewei Decoction could decrease visceral hypersensitivity by downregulating calcitonin gene-related peptide and substance P expression in the esophageal mucosa [39] and downregulating ncNOS and c-Fos expression in the brain and spinal cord dorsal horn of rats [40]. Meanwhile, in our meta-analysis, we found that the formulas in almost all the included studies contained soothing liver (zang-fu organs in TCM) herbs, which are herbs with emotional regulation (Table 3). In these studies, the total effective rate of TCM was higher than that of Western medicine. Therefore, we believe that the important mechanism of TCM in the treatment of NERD may decrease the visceral hypersensitivity to improve symptoms.

The meta-analysis demonstrated the effectiveness of TCM therapy alone for NERD patients as compared with the conventional Western medicine. For the clinical efficacy, we found that the total effective rate of TCM alone was superior to single PPIs or Prokinetics; nevertheless, it was equivalent to the therapeutic combination of PPIs and Prokinetics. This finding showed that the effectiveness of TCM alone was better than PPIs or Prokinetics alone in improving outcomes in NERD patients. One evident difference with Western medicine is that TCM therapy has two main characteristics, including a holistic view and Syndrome Differentiation Treatment, which can provide a personalized therapy based on symptoms of the patient by treating the patient as a whole. Patients with NERD are clinically inclined to overlap with symptoms of functional dyspepsia, irritable bowel, and functional constipation, such as persistent abdominal fullness, persistent or intermittent diarrhoea, and constipation. Taking Western medicine, such as PPIs and Prokinetics, cannot improve these overlapping symptoms; instead, the dialectical treatment of TCM can provide a good effect [41], potentially by promoting gastrointestinal motility. Furthermore, animal experiments have shown that TCM can lower the gastric acid levels, raise the level of plasma motilin, and increase the content of pepsin in gastric juice in rats [42]. Accordingly, we propose that TCM is able to provide an effective, extensive, and novel mind.

RDQ is currently the world's most recognized and widely used diagnostic GERD-specific scale. RDQ is a medical history survey mainly based on symptom scores, and its effectiveness and reliability in the diagnosis of GERD have been confirmed at home and abroad [43, 44]. Without endoscopic evidence of esophageal mucosal damage, in general, an RDQ score ≥ 12 is one of the diagnostic criteria of NERD and is an essential inclusion criterion [45]. When RDQ score is <12, it indicates that symptoms of NERD have disappeared or clearly improved. Therefore, we selected RDQ as one of the secondary outcomes. From the result of the meta-analysis, TCM alone had more advantages over Western medicine in improving RDQ symptoms, even though significant heterogeneity existed.

As shown in the meta-analysis, the recurrence rate in the TCM group was relatively lower than in the Western medicine group. Clinical trials have validated that TCM can lead to better symptomatic remission and a lower relapse rate in comparison with Western medicine [46]. A clinical trial found that the efficacy of Banxia Houpu Decoction with Zuo Jin Pill in NERD was comparable to that of domperidone combined with omeprazole (control group), and the relapse rate at 12 weeks after ceasing treatment was signally lower in the TCM group [47]. The pathogenesis of NERD involves a variety of factors, including gastric reflux (acidic, alkaline, and bile reflux), esophageal motility abnormalities, esophageal mucosal hypersensitivity, and psychiatric disorders [48]. TCM therapy, a multitarget, multilevel, and coordinated intervention effect against NERD, can effectively reduce the recurrence rate.

The meta-analysis summarized evidence on the adverse events in the two groups and found that a single application of TCM produced fewer side effects than Western medicine. Due to the lack of a radical cure, long-term treatments are required for NERD patients, and PPIs use is associated with the increased risk of side effects. Many clinical studies regarding TCM with NERD patients have reported rare side effects, perhaps because the majority of Chinese medicine practitioners choose nontoxic herbs to treat NERD patients.

However, this meta-analysis had the following limitations. First, the majority of included studies failed to make blinded assessments, and only one study [13] reported the double-blind and double-dummy method, which may have influenced the objectivity of NERD outcomes. Second, the inclusion criteria of these studies in recruiting patients were inconsistent and nonstandard. Most trials chose the clinical effect as the first outcome, whereas eight excluded trials selected the symptom relief rate. Therefore, we could not analyze these data and removed the eight studies. Third, most included studies had small samples sizes with mid-to-low-quality designs, possibly exerting an impact on the outcomes and publication bias. Due to low quality, trial groups receiving therapeutic combinations of TCM and Western medicine, and failure to meet the inclusion criteria, these studies were excluded from the meta-analysis. To overcome the above-mentioned limitations, high-quality, well-designed, large sample trials focused on the efficacy and safety of TCM therapy for NERD should be performed in the future.

## 5. Conclusion

This meta-analysis provides evidence that TCM therapy alone can improve the relief of NERD symptoms, decrease the recurrence rate, and reduce adverse events, which is better than PPIs or Prokinetics alone possibly through multitarget and multilevel intervention effects. For NERD treatment, TCM alone or in combination with Western medicine, such as PPIs, will be more effective. TCM is expected to be a promising alternative therapy for NERD patients in the future.

## Figures and Tables

**Table 1:** Evaluation criteria on the efficacy of TCM symptoms and syndromes recommended by GCRNDTCM.

Classification	Detailed description
Cure	Clinical symptoms and signs completely disappeared
Markedly	Clinical symptoms and signs disappeared or were significantly reduced, with a total score ratio reduction of 2/3 or more
Effective	Clinical symptoms and signs were partially reduced, with a total score ratio reduction of 1/3 or more
Invalid	Clinical symptoms and signs had no significant change, with total score ratio reduction of 1/3 or less

TCM, traditional Chinese medicine; GCRNDTCM, Guidelines of Clinical Research of New Drugs of Traditional Chinese Medicine; total score ratio = (pretreatment total score − posttreatment total score)/pretreatment total score *∗* 100%.

**Table 2:** Baseline characteristics of studies included in the meta-analysis.

Study	Sample size (T/C)	Sex (M/F)		Age		TCM intervention	Control regimen	Treatment duration (weeks)	Main outcomes	Randomized method
		T	C	T	C					
Cheng et al. 2013 [9]	30/30	12/18	7/23	50.47 ± 11.62	46.63 ± 12.40	Shugan Hewei Decoction, (1 dose/d)	Ome (40 mg/d)	8	TER	RNT

Fu and Li 2016 [10]	59/59	32/27	30/29	39.20 ± 10.80	41.70 ± 11.60	Ningshen Qingdan decottion (1 dose/d)	Mos (15 mg/d) and Eso (40 mg/d)	8	TER, RDQs	RNT

Huang et al. 2010 [11]	100/100	63/37	55/45	42.80 ± 11.40	38.70 ± 10.80	Guanyanling Granule (30 g/d)	Ome (40 mg/d)	4	TER	RNT

Li 2006 [12]	25/25	8/17	9/16	46.50 ± 13.60	45.80 ± 14.20	Huanglian Wendan Decoction (1 dose/d)	Gas (40 mg/d) and Mot (30 mg/d)	4	TER, RDQs	RNT

Li et al. 2011 [13]	57/55	30/27	30/25	50.91 ± 10.47	46.86 ± 14.22	Tongjiang Granule (30 g/d)	Mos (15 mg/d)	4	TER, RDQs	Randomized block

Li et al. 2013 [14]	58/56	29/29	33/23	50.70 ± 10.60	47.10 ± 13.30	Tongjiang Granule (30 g/d)	Ome (40 mg/d)	4	TER, RDQs	Sealed envelopes

Pan and Liu 2014 [15]	30/30	11/19	8/22	45.46 ± 13.59	46.78 ± 12.48	Qingdan Hewei decoction (1 dose/d)	Panto (80 mg/d)	6	TER	RNT

Wang and Deng 2013 [16]	45/45	23/22	21/24	48.10 ± 6.30	47.20 ± 7.40	SiNi pulvis (1 dose/d)	Eso (40 mg/d) and Mot (30 mg/d)	4	TER, RDQs	RNT

Yang et al. 2013 [17]	64/63	21/43	17/46	50.42 ± 10.01	46.27 ± 12.19	Banxia Xiexin Decoction (1 dose/d)	Panto (40 mg/d)	8	TER, RDQs	RNT

Yang and Liu 2014 [18]	50/50	27/23	28/22	22–63	22–61	Jiangni Huatan Yiqi Hewei method (1 dose/d)	Eso (30 mg/d) and Talcid (1-2 tablet/d)	4	TER	RNT

Zhang 2009 [19]	30/30	17/13	15/15	38.6 ± 10.6	37.70 ± 11.50	Guanyanling Granule (30 g/d)	Ome (40 mg/d)	4	TER, RDQs	RNT

Zhang et al. 2012 [20]	37/36	15/22	15/21	39.23 ± 9.8	40.01 ± 7.78	Jianpi Jiangni Decoction (1 dose/d)	Rabe (20 mg/d)	4	TER, RDQs	Sealed envelopes

Zhong et al. 2011 [21]	80/80	49/31	46/34	32.9 ± 18.6	34.50 ± 11.30	Jiangni Hewei Decoction (1 dose/d)	Rabe (20 mg/d)	8	TER	RNT

Zhou et al. 2012 [22]	60/60	35/25	38/22	41.8 ± 12.2	42.10 ± 11.40	Shensang Banfo Decoction (1 dose/d)	Rabe (20 mg/d) and Mos (15 mg/d)	8	TER	RNT

T, TCM group; C, control group; NA, not available; Ome, omeprazole; Mos, mosapride; Eso, esomeprazole; Gas, gaster; Mot, motilium; Panto, pantoprazole; Rabe, rabeprazole; TER, total effect rate; RDQs, Reflux Disease Questionnaire score; RNT, random number tab.

**Table 3:** Herbal medicines soothing liver in most included studies.

References	Formula	Herbs soothing liver
Cheng et al. 2013 [9]	Shugan Hewei Decoction	Radix Bupleuri; Fructus Toosendan
FuandLi 2016 [10]	Ningshen Qingdan decottion	Radix Bupleuri; Cyperus rotundus; Radix Bupleuri; Fructus Toosendan; Curcuma Aromatica
Huang et al. 2010 [11]	Guanyanling Granule	Pinellia Tuber
Li et al. 2011 [13]	Tongjiang Granule	Cyperus rotundus; Evodia Rutaecarpa
Li et al. 2013 [14]	Tongjiang Granule	Cyperus rotundus; Evodia Rutaecarpa
Pan and Liu 2014 [15]	Qingdan Hewei decoction	Radix Bupleuri; Radix Bupleuri; Fructus Toosendan; Curcuma Aromatica
Zhang 2009 [19]	Guanyanling Granule	Radix Bupleuri; Fructus Toosendan
Zhang et al. 2012 [20]	Jianpi Jiangni Decoction	Radix Bupleuri; Radix Paeoniae Alba; Zuo Jin pill
Zhong et al. 2011 [21]	Jiangni Hewei Decoction	Radix Bupleuri; Radix Bupleuri; Fructus Toosendan
Zhou et al. 2012 [22]	Shensang Banfo Decoction	Finger Citron
